# Climate-Determined Suitability of the Water Saving Technology "Alternate Wetting and Drying" in Rice Systems: A Scalable Methodology demonstrated for a Province in the Philippines

**DOI:** 10.1371/journal.pone.0145268

**Published:** 2015-12-21

**Authors:** Andrew Nelson, Reiner Wassmann, Bjoern Ole Sander, Leo Kris Palao

**Affiliations:** 1 Faculty of Geo-Information and Earth Observation (ITC), University of Twente, Enschede 7500 AE, The Netherlands; 2 International Rice Research Institute (IRRI), Los Baños 4031, Philippines; Wageningen University, NETHERLANDS

## Abstract

70% of the world’s freshwater is used for irrigated agriculture and demand is expected to increase to meet future food security requirements. In Asia, rice accounts for the largest proportion of irrigated water use and reducing or conserving water in rice systems has been a long standing goal in agricultural research. The Alternate Wetting and Drying (AWD) technique has been developed to reduce water use by up to 30% compared to the continuously flooded conditions typically found in rice systems, while not impacting yield. AWD also reduces methane emissions produced by anaerobic archae and hence has applications for reducing water use and greenhouse gas emissions. Although AWD is being promoted across Asia, there have been no attempts to estimate the suitable area for this promising technology on a large scale. We present and demonstrate a spatial and temporal climate suitability assessment method for AWD that can be widely applied across rice systems in Asia. We use a simple water balance model and easily available spatial and temporal information on rice area, rice seasonality, rainfall, potential evapotranspiration and soil percolation rates to assess the suitable area per season. We apply the model to Cagayan province in the Philippines and conduct a sensitivity analysis to account for uncertainties in soil percolation and suitability classification. As expected, the entire dry season is climatically suitable for AWD for all scenarios. A further 60% of the wet season area is found suitable contradicting general perceptions that AWD would not be feasible in the wet season and showing that spatial and temporal assessments are necessary to explore the full potential of AWD.

## Introduction: Irrigation for field crops

### Quantity and inefficiency

Irrigated agriculture is one of the major consumers of freshwater accounting for 70% of water withdrawal globally [1,2]. At sub-continental scale, these percentages can be even higher, e.g. 92% in mainland Southeast Asia and 72% in maritime Southeast Asia [1]. To meet growing food demand, the amount of water used for producing food and fodder crops is expected to increase further at a rate of 0.7% per year [3]. More than 50% of the world’s 271 million ha of irrigated crop area is located in Asia where rice production accounts for 40–46% of the net irrigated crop area [4].

The spread of irrigation has substantially contributed to increased yields obtained during the Green Revolution, but the annual growth rates in irrigated area have been very low in recent decades [5]. While water scarcity is aggravating in many countries, it can be anticipated that competition with other water uses will limit availability of water for irrigated agriculture. Moreover, climate change will increase uncertainties for obtaining irrigation water when and where needed [6].

Water-saving technologies such as drip and pivot irrigation have been used in upland (non-flooded) crops, but have limited suitability for paddy rice production. At field level, rice receives up to 2–3 times more water than other irrigated crops [7], but an unknown proportion of the water losses from individual fields is reused by other fields downstream. Discounting for this reuse, it can be estimated that irrigated rice receives some 34–43% of the total world’s irrigation water, or 24–30% of the total world’s freshwater withdrawals [7].

However, irrigation water is often used inefficiently and in many cases used in an unsustainable manner [8]. Depending on the actual source of water, irrigation can be classified into (i) surface irrigation and (ii) groundwater pumping. Many surface irrigation schemes in Asia consist of decades-old canal infrastructure that are often degraded due to insufficient maintenance [9]. Thus, water supply in many irrigation schemes of Asia is impaired by substantial losses that accrue between the reservoir or river diversion and the end user. As for groundwater pumping, the supply chain is much shorter, so that water losses are lower. On the other hand, this system requires substantial energy input and can lead to detrimental effects on the ecological functionality of the aquifers [8].

In the Philippines, the actual renewable freshwater resources are in the range of 480,000 km3 per year while the irrigation water withdrawal accounts for 65,590 km3 per year (data from 2006) [10]. This corresponds to 13.7% of the freshwater resources used for irrigation which is a higher percentage than for other countries of SE Asia, e.g. Thailand (11.8%), Vietnam (8.8%) and Indonesia (4.6%) [10].

### Solutions to scarcity

The growing freshwater scarcity is already evident in many parts of the world, so that raising water productivity in agriculture (“more crop per drop”) is key for reducing pressure on the global freshwater resources [11,12]. Reducing the amount of irrigation water needed for rice production has been a long-standing goal of agricultural research and development. Several rice growing countries have embarked on programs to upscale improved irrigation techniques starting in the 1980s. While these studies have initially focused on areas, with extensive water shortage such as parts of China and India, water studies starting in 1990’s have included irrigation systems where water scarcity is a seasonal or spatially contained problem [13].

As a response to declining water availability for rice, the International Rice Research Institute has developed the Alternate Wetting and Drying (AWD) technique in close partnership with national research institutions. The principle of AWD is to switch from a continuously flooded rice field to a field that encompasses several dry phases during the growing season. Starting at about 2–3 weeks after transplanting (3–4 weeks after sowing) the field is left to dry out until the water table reaches a level of about 10–15cm below the soil surface. Depending on the soil, this may take between 1 day and more than 10 days given that no precipitation occurs. Once the threshold is reached, irrigation water should be applied until 3–5cm of standing water in the field is reached. A perforated plastic or bamboo tube that is sunk into the soil allows checking the below-ground water table in order to irrigate at the right time [14]. A level of “-15cm” has been identified as ‘safe’ so that plants don’t face drought stress and thus yields are not reduced [7]. This irrigation technique reduces water use by up to 30% without impacting yield [15,16]. AWD has been widely tested and is now being propagated in many Asian countries with large scale adoption in the Philippines, Vietnam, and Bangladesh [16]. At the same time, adoption of AWD by farmers can cause major challenges in irrigation areas where farmers get sufficient water from an irrigation scheme without a volume-dependent price attached to it [17,18].

In addition to water saving, AWD also reduces emissions of the Greenhouse Gas methane [19]. In fact, the dissemination efforts for AWD have recently gained large momentum because it is ranked as the most promising mitigation option in rice production while the water saving aspect is regarded more as incentive to promote this technology [20]. The growing awareness and recognition of AWD as a promising mitigation option is reflected in recent policy decrees, e.g. Vietnam’s Ministry of Agriculture and Rural Development aiming a dramatic expansion until 2020. The approach also has been incorporated into international regulations on the Clean Development Mechanism, namely (i) through an approved baseline methodology [21] and (ii) definition of standardized baselines at national levels, e.g. in the Philippines [22].

### Aim: Assessing the viability of AWD as a mitigation option for rice

Mitigation options like AWD that require changes in national policy for successful implementation need to be guided by quantitative assessments of their viability. No such assessment has been made for AWD on national or even larger scales. Ideally such assessments should rely on a standard method as well as consistent and comparable information that is available in any rice growing country, but still be flexible enough to include country specific requirements where needed.

This study is the first to propose a standard method that can be applied in any rice growing country to provide an upper bound of the potential area for AWD in rice systems.

The aim of this study is to develop and test a spatially explicit water balance model to estimate the area and seasons that are climatically suitable for AWD in rice. It should be noted though, that the term ‘suitability’ in the context of this study only captures the water balance without irrigation. Hence this methodology defines an upper bound of AWD suitability that is based on climate limiting factors that are applicable to any rice growing area or system. There will be other aspects of suitability, such as the availability and the necessity of pumping and the ability to drain within the topography that can be incorporated into this method for more comprehensive and targeted AWD suitability assessments in the future.

We first outline our data requirements and methodology for the AWD suitability assessment, the sensitivity analyses that were used to address data uncertainty and give a description of the study site of Cagayan province in the Philippines. Results of the AWD suitability assessment and sensitivity analysis are then presented and discussed. We conclude with a proposal to scale up such assessments to national and regional scales.

### 2 Materials and methods for a climate determined suitability of AWD

**Fig 1:**
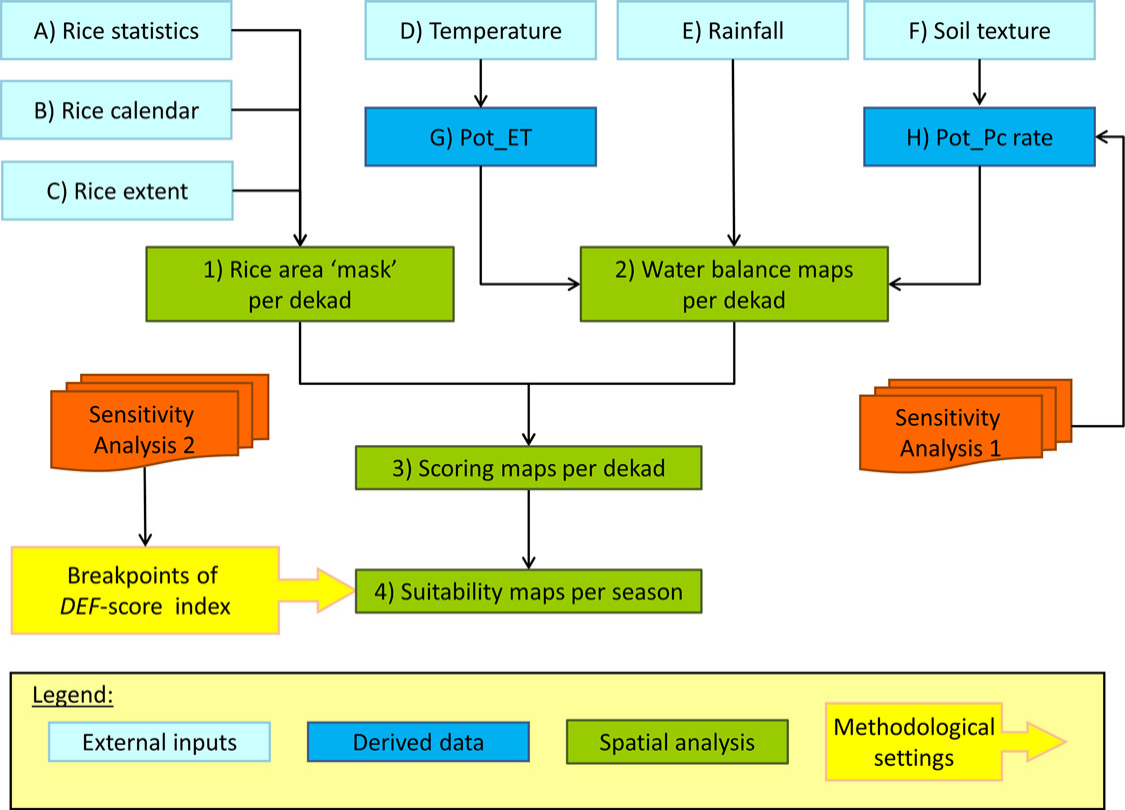
Flow chart of the AWD suitability analysis.

Fig 1 describes the data requirements, information flow and sensitivity analyses for the suitability assessment, and will be referred to throughout this section. The analysis estimates the locations, area and seasons that are climatically suitable for AWD. Different spatial datasets have been prepared for this suitability analysis as applied to Cagayan province, Philippines, a major rice producing province of the country.

### Study area and input data

#### Study area

**Fig 2:**
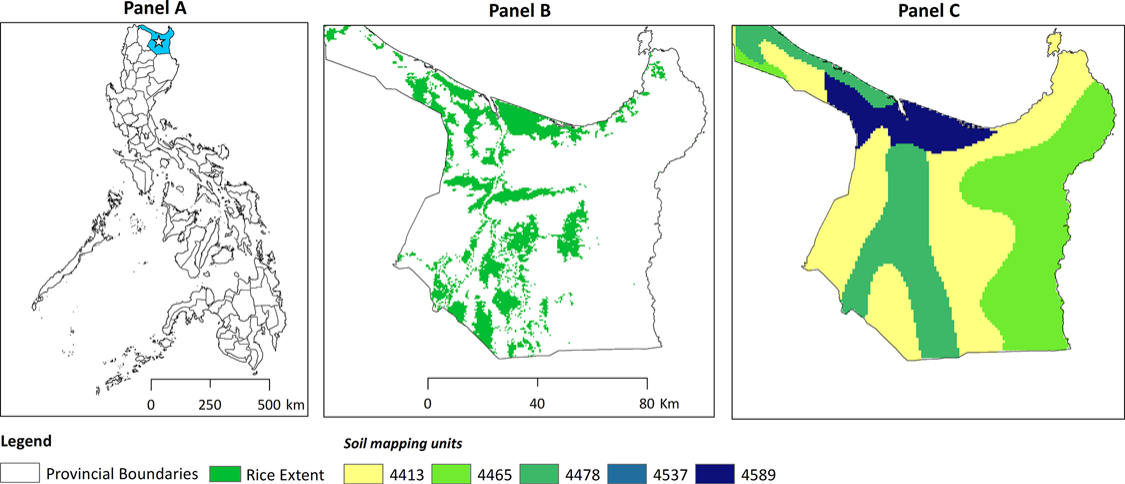
The Philippines and key spatial datasets for Cagayan province. Panel A: The Philippines, with Cagayan province highlighted. Panel B: Extent of the rice growing area in Cagayan province: Panel C: Soil Mapping Units (SMU) in Cagayan province derived from the Harmonized World Soil Database (HWSD).

Cagayan province is situated in the north of Luzon island in the Philippines (Fig 2A). In this study we exclude the Babuyan islands to the north and focus on the main area of the province on Luzon covering 8,200 square kilometres between 120.95E and 122.36E and 17.48N and 18.68N. The 2010 census estimates the population at 1.12 million [23]. The province’s geography is characterised by the Cagayan river (also called Rio Grande de Cagayan) flowing from south to north and discharging into the Babuyan channel and South China Sea. It is the longest and largest river in the Philippines leading to substantial deposits of clay along the Cagayan valley. The valley is bounded by the Cordillera mountains to the west and the Sierra Madre range in the east. The most important crops are rice (Fig 2B), maize (over 300,000 hectares combined), banana, sugarcane, coconut, mango and tobacco (around 90,000 hectares combined) [24]. Annual rainfall is 1350 mm, with most rain (950 mm) occurring during the monsoon season from May to October. The climate is semi-tropical and temperature is relatively stable through the year with a minimum of 23°C and maximum of 34°C. Rice is cultivated under both rainfed (22% of the rice area) and irrigated (78% of the rice area) conditions and can be grown twice a year in the monsoon or wet season and dry season.

#### External input data

The assessment relies on spatial analysis of the water balance in rice growing areas on a regular time-step through the rice growing season using data (Table 1) that either are available or are likely to be available in any rice growing country in Asia. Hence the first requirement is information on where and when rice is cultivated (Fig 1, External inputs A, B and C) so that the analysis is constrained to rice growing areas and rice growing seasons.

**Table 1:** Overview of external data sets used in the suitability assessment.

Parameter	Source	Unit of measurement, spatial and temporal resolution
Data set A Rice statistics	Philippine Statistics Authority Quarterly Harvested Rice Area Estimates [24]. Province boundaries from [25].	Hectares. Province level. Quarterly estimates for 2012.
Data set B Rice calendar	Rice crop calendar database [26].	Day of year. Province level. Typical planting and harvesting dates to the nearest 15 days.
Data set C Rice extent	Rice extent map of the Philippines [27].	500m raster of rice extent between 2000–2012.
Data set D Temperature	Daily minimum and maximum temperature from Global Surface Summary of the Day (GSOD) station data [28] interpolated to match the spatial resolution of the Tropical Rainfall Measuring Mission (TRMM) data.	Degrees C. 0.25 degrees (~28km) raster. Daily values (2003–12) aggregated to 10-year means per dekad.
Data set E Rainfall	NASA Tropical Rainfall Measuring Mission, TRMM [29].	mm/d. 0.25 degrees (~28km) raster. Daily rainfall values (2003–12) aggregated to 10-year means per dekad.
Data set F Soil texture	Harmonized World Soil Database [30].	Soil Mapping Unit (SMU). 30 arc seconds (~1km) raster. No temporal information.

Data set A. Harvested rice area statistics for Cagayan province for each quarter in 2013 were acquired from the Philippine Statistic Authority [24]. Quarterly estimates of harvested rice area in hectares for 2012 were Q1 = 61,379, Q2 = 63,681, Q3 = 31,288, Q4 = 60,230. This tabular data was linked to provincial boundaries from the Global Administrative Areas Database [25].

Data set B. Peak planting and harvesting dates for the wet and dry seasons were derived from a rice crop calendar for the Philippines [26]. Peak refers to the date when the majority of the crop is being planted or harvested, respectively. The rice calendar database records these dates to the nearest bi-monthly interval. For the wet season, peak planting and harvesting dates are May 15 and September 15 respectively. For the dry season, peak planting and harvesting dates are December 15 and April 15 respectively. These dates were compared to the quarterly harvested rice areas from Data set A to estimate the rice area for each reported season which were Q1 and Q2 for the dry season (125,060 ha) and Q3 and Q4 for the wet season (91,518 ha).

Data set C. Data sets A and B provide the provincial overview of how much rice is grown but they do not provide spatial information of where rice is grown within the province. To provide the required level of spatial detail on rice area we used a rice extent map that shows the spatial distribution of rice based on a temporal analysis (2000–2012) of MOD09A1 data using a previously published paddy mapping algorithm [31]. MOD09A1 is a remote sensing product derived from the Moderate Resolution Imaging Spectrometer (MODIS) instrument on board the Terra and Aqua satellites. The MOD09A1 product provides surface reflectance 8-Day global composites of seven bands of surface reflectance at 500 meter resolution. Each product pixel contains the best possible observation during an 8-day period as selected on the basis of high observation coverage, low view angle, absence of clouds or cloud shadow, and aerosol loading. This product provides regular spatial observations of the earth’s surface than can be interpreted and classified into different land covers categories. MODIS data is freely available in a 10 degree by 10 degree tile system that covers the globe. Tile h29v07 covers Cagayan province.

The algorithm was slightly modified to exclude wetlands and aquaculture areas [27]. We ran the algorithm over 13 years of MOD09A1 data for tile h29v07 and merged the results from each year to provide a single 500m resolution, rice extent map that captured the maximum extent of rice in recent years (Fig 2B). A longer time series was used for this data than for others in this to mitigate the effect of pervasive cloud cover which limits the rice detection skill from year to year.

The water balance component of the climate-determined suitability assessment (Fig 1, Spatial analysis 2) relies on weather data and soil characteristics that define the amount of water entering and leaving the system on a dekad time-step. The input data for this component are temperature, rainfall and soil texture (Fig 1, External inputs D, E and F). The weather data need to be available on a regular time-step and we identified three criteria that would determine the appropriate choice of time-step:

- Water management decisions (to irrigate or drain a field) typically take place on a weekly or fortnightly basis.
- Climate is often referred to on a weekly, monthly or annual time-step.
- Most rice varieties in Asia mature between 90 (short duration) and 150 (long duration) days with 110–120 days being the average.

To ensure sufficient number of data points and following previously published examples for rainfall data, we selected a dekadal time-step for the temporal aggregation of weather data.

Data set D. Temperature data in °C were derived from daily minimum and maximum temperature data from weather stations in the Global Surface Summary of the Day (GSOD) database [28]. Daily station data from 53 reporting stations in the Philippines for 2003–2012 were interpolated onto a regular grid to match the spatial resolution of the rainfall data in Data set E. Two of these stations are in Cagayan province: Aparri at 121.62E, 18.36N and Tuguegarao at 121.73E, 17.61N. The interpolation used elevation as a covariate in a thin-plate-spline interpolation algorithm. These daily minimum and maximum temperature rasters were averaged to give the mean daily temperature which was in turn averaged to give the mean temperature per dekad for all dekads (2003–12). These dekadal temperatures were again averaged to give the 10 year mean temperature per dekad (2003–12).

**Fig 3:**
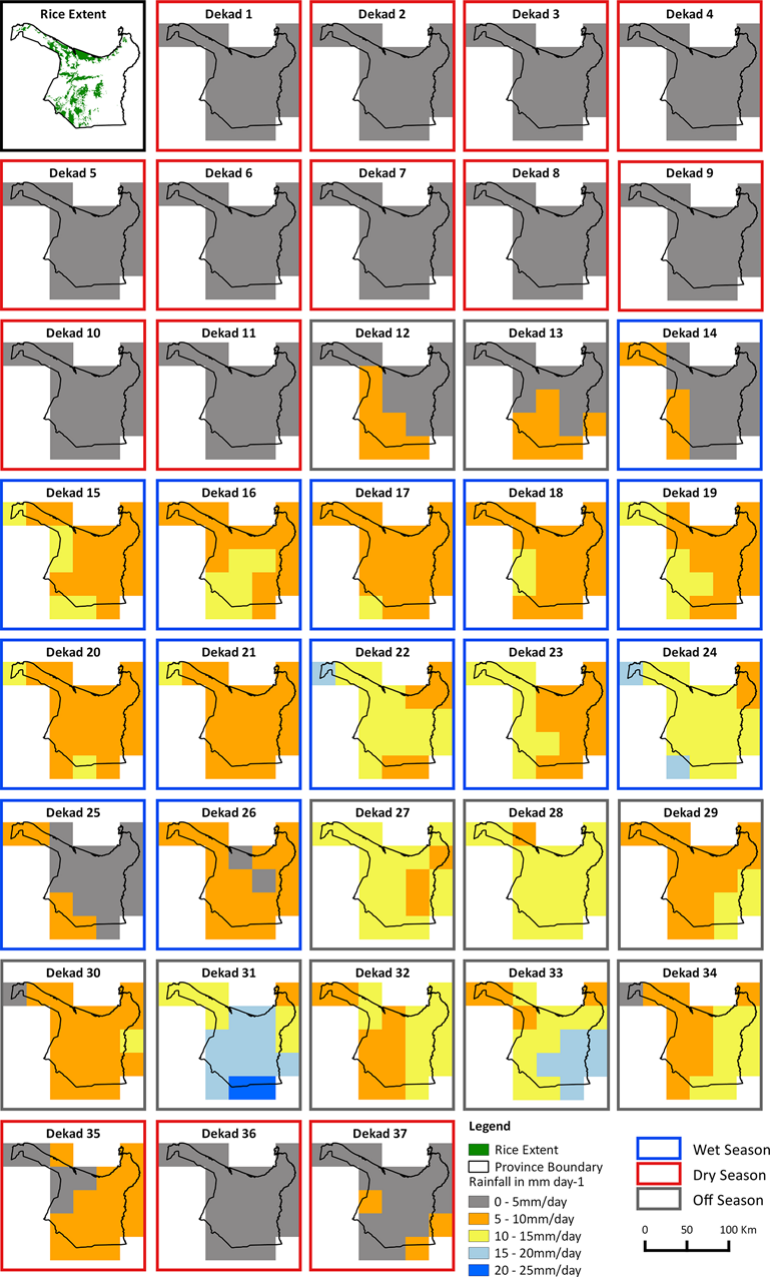
10 year mean rainfall in mm/dekad. Top left panel shows the rice extent map as a reference, then sequentially from left to right, top to bottom the remaining 37 panels show the spatial distribution of the 10 year mean daily rainfall per dekad. The border of each rainfall map shows if the dekad is in the dry season (red), wet season (blue) or off-season (gray).

Data set E. Rainfall data in mm/d were derived from the Tropical Rainfall Measuring Mission (TRMM) database that provides daily rainfall data from 50°N to 50°S in raster grid format with 0.25 degree (~28km) resolution [29]. We summed daily rainfall (from the calibrated daily 3B42 product) per dekad for all dekads (2003–2012) and then computed the 10 year mean rainfall per dekad (2003–2012). Fig 3 shows the spatial distribution of the 10 year mean rainfall per dekad.

Data set F. Soil texture data were extracted from the Harmonized World Soil Database (HWSD) [30]. The HWSD raster layer has a spatial resolution of 1km and is based on a standardized interpretation of many soil maps and soil reference datasets resulting in a global soil database. The HWSD contains spatial soil mapping units (SMU) which are composed of dominant and associated soils. A SMU can be composed of one or more soils, with three or four soils per SMU being typical. Each soil type has a defined texture class which can be associated with quantitative estimates of seepage and percolation rates. The relative area of each soil type within the SMU is known but the exact spatial distribution within the SMU is unknown. Fig 2C shows the SMU codes for Cagayan province and Table 2 shows the composition of soils per SMU.

**Table 2:** Soil types and their share within each soil mapping unit in Cagayan province.

		Soils and share per soil mapping unit		
SMU code	Area per SMU (sq km)	Clay (light)	Loam	Sandy clay loam
4413	3,495	20	60	20
4465	2,701	20	60	20
4478	1,761	20	80	0
4589	681	50	50	0
4537	361	20	60	20

#### Derived input data

**Fig 4:**
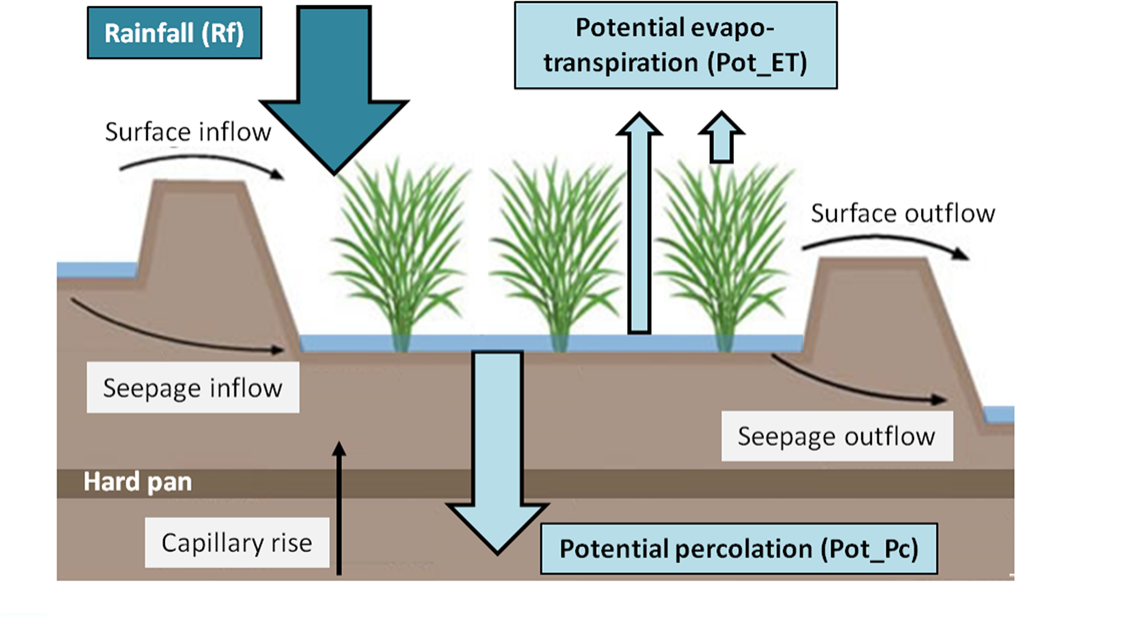
Schematic display of the water balance in flooded rice fields without irrigation (redrawn with modifications after [32]). Blue (thick) arrows in the figure are the only water fluxes considered in the water balance calculations. Black arrows show other water flows that are not considered.

Rainfall is the single source of water gain in our water balance model (Fig 4), but two additional datasets (Table 3) needed to be derived to estimate the major sources of water loss (Fig 1, Derived data G and H).

**Table 3:** Overview of derived data sets used in the suitability assessment.

Parameter	Computation	Unit of measurement, spatial and temporal resolution
Data set G Potential evapotranspiration	Hargreaves method	mm/day. 0.25 degrees (~28km) raster: daily values aggregated to 10-year means per dekad
Data set H Potential percolation	(i) As a function of texture, (ii) Fixed values	mm/day. 30 arc seconds (~1km) raster: no temporal information

**Fig 5:**
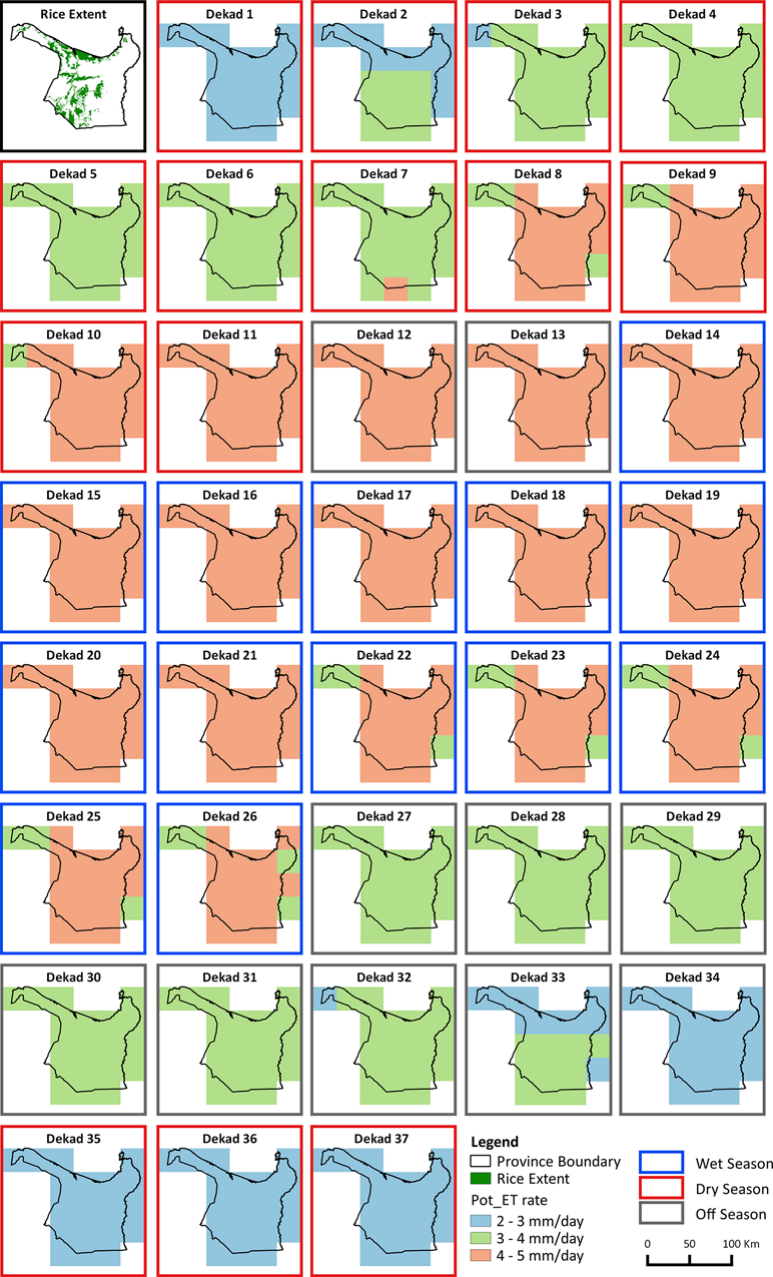
10 year mean Pot_ET in mm/dekad. Top left panel shows the rice extent map as a reference, then sequentially from left to right, top to bottom the remaining 37 panels show the spatial distribution of the 10 year mean Pot_ET per dekad. The border of each Pot_ET map shows if the dekad is in the dry season (red, wet season (blue) or off-season (gray).

Data set G. Potential evapotranspiration (Pot_ET) in mm/d is one source of water loss in our simple water balance assessment (Fig 4). Pot_ET or even ET is not readily available from sufficient numbers of weather stations in the Philippines and must be derived from other methods. Following the recommendation of [33] we selected the Hargreaves method [34] to derive Pot_ET, which uses temperature and extra-terrestrial radiation (Eq 1),

$$Pot\_ET=0.0023Ra(Tmean+17.8)TD^{0.5}$$ (1)

where *0*.*023* is the Hargreaves coefficient, *Ra* is the extraterrestrial radiation in mm/d, *Tmean* is the average temperature in °C and *TD* is the daily temperature range in °C. We used the approach in [35] to compute *Ra* for each day of the year and for different latitudes (Eq 2),

$$Ra=0.408\frac{24(60)}{\pi }G_{sc}d_{r}[\omega_{s}\sin(\phi )\sin(\delta )+\cos(\phi )\cos(\delta )\sin(\omega_{s})]$$ (2)

where *0*.*408* is the inverse latent heat flux, *G*_*sc*_ is the solar constant, d_r_ is the inverse relative distance between the Sun and the Earth, *ω*_*s*_ is the sunset hour angle, *ϕ* is the latitude, and *δ* is the solar declination. *Ra* was computed on a daily time step per 0.25 degrees of latitude and used, with daily temperature data (as in data set D) to compute a daily estimate of Pot_ET. These daily estimates were then summed per dekad and used to create an average Pot_ET per dekad during 2003–2012. Fig 5 shows the spatial distribution of the 10 year mean Pot_ET per dekad.

Data set H. There are horizontal and vertical water movement in the rice field soils. Water enters and leaves the rice field through seepage (inflow and outflow), capillary movement and percolation (Fig 4). At field scale, we assumed that the lateral inflows and outflows (i.e. seepage) are equal and that the capillary movement is only a minor fraction that can be neglected in the overall water balance equation. Thus, our assessment focused on the vertical losses in form of percolation. However, it should be noted that percolation rates determined for rice production systems refer to flooded conditions and thus, represent the upper limit of the rate of percolation into the subsoil. Thus, we adopted the concept of ‘potential percolation’ (Pot_Pc) as developed by [36] for a hydrological model of rice fields.

Percolation rates are influenced by the range of characteristics pertaining to the physical and hydraulic properties of the soil. The correlation between texture and permeability is well established for upland soils, but rice soils typically develop a ‘hard pan’ as a result of repeated puddling. This hard pan will reduce percolation and also create a degree of uniformity among different texture classes in terms of permeability rates. Nevertheless, the impact of texture is still observable, i.e. sandy soils have a higher percolation than clay soils irrespective of an existing plow pan.

[37] reported percolation rates of well-puddled soil in the range from 2.6 to 3.6 mm day^-1^ that corroborated earlier values measured by [38] and are deemed typical for silty clay rice soils. However, percolation rates will be much higher in sandy soils; [39] reported rates of 13 mm/d even when these soils have thoroughly been puddled. Typical combined values for seepage and percolation vary from 1–5 mm/d in heavy clay soils to 25–30 mm/d in sandy and sandy loam soils [40].

These uncertainties surrounding the permeability of rice soils are addressed here in a sensitivity analysis (Fig 1, Sensitivity Analysis 1). We took two approaches to address the uncertainty in Sensitivity Analysis 1, one where the range of plausible percolation rates were dictated by soil texture, and another where we hypothesised that hard pan development and other factors (such as uncertainty in the geo-location of specific soil types within the SMU) will reduce the variability in percolation across soil types.

For the first approach, we set a range of Pot_Pc values for different texture classes of rice soils. We determined Pot_Pc rates as a function of soil texture as shown in Table 4. The rates for sand are shown for completeness, though this soil texture is not present in the SMUs for Cagayan province. The soil texture specific values encompassed the upper and lower ranges of texture specific Pot_Pc rates. These Pot_Pc rates were applied to the soil texture classes in data set F. Since the distribution of each soil within the SMU is unknown we estimated the SMU-specific Pot_Pc rate based on the average Pot_Pc across all soil textures within that SMU, weighted by the proportional area of each soil texture within the SMU. Our second approach used fixed Pot_Pc values for the entire province of 1, 2, 3, 4, 5, 10, and 15 mm/d.

**Table 4:** Potential percolation rates as a function of soil texture.

Soil texture class	Ranges of Pot_Pc values mm/d		
	Basic setting	Lower bound	Upper bound
Clay (light)	3.0	1.0	5.0
Loam	4.0	1.2	6.0
Sandy clay loam / sandy loam	9.0	2.9	15.0
Sand	12.0	5.0	20.0

The upper values for Pot_Pc rates are somewhat less than those reported in the literature, e.g. the Rice Almanac mentions 25–30 mm/d in sandy and sandy loam soils [40]. However, we felt that we should omit such high values, because it seems likely that such high outflows from one field will eventually lead to an inflow into another field through seepage. Thus, the net-balance for a given field may be less than these very high (gross) Pot_Pc rates. In maintaining relatively low Pot_Pc values even in the upper range, we tried to remain on the ‘conservative’ side for classifying rice fields as suitable or highly suitable for AWD.

### Spatial analysis for the suitability assessment

The four spatial analysis steps from Fig 1 are outlined below.

Step 1. Rice area ‘mask’ per dekad. Data set A provides the harvested rice area per season and Data set B provides the start and end dates of the season. Data set C provides the spatial distribution of the maximum rice extent (the spatial union of the rice extent across both wet and dry seasons) within the province on a 500m pixel basis. The following assumptions and procedures were followed to generate this rice area mask.

Data set B was temporally disaggregated from a 15 day time step to dekadal time steps where the observed planting or harvesting dates were assigned to their respective dekad. The harvested area per season per province from Data set A was then assigned to those dekads resulting in 37 (36 dekad maps, plus one additional ‘dekad’ map of the last 5 or 6 days of the year) maps of the rice area per province. The final step is to distribute this provincial rice area per dekad to the physical rice extent pixels in Data set C on an equal area basis (the rice area in the province for that dekad is distributed equally across all rice pixels in Data set C). The result is 37 maps at 500m resolution that can be used to estimate the rice area that meets the climatic suitability criteria per dekad, per season and per year.

Step 2. Water balance maps per dekad. The basic assumption of this climate-dependent suitability assessment is that the ability–as well as the necessity–to drain the rice field will depend on the actual water balance (Fig 4). High rainfall will obviously thwart the suitability for AWD. 37 water balance maps at 0.25 degree (about 28km) resolution, were computed by subtracting Pot_ET and Pot_Pc from Rf on a dekad time-step.

Step 3. Scoring maps per dekad. Having determined the water balance score for each pixel and each dekad we scored it according to a deficit (DEF) or excess (EXC) water balance.

WaterbalancedeficitifRf<Pot_ET+Pot_Pc (3)

WaterbalanceexcessifRf≥Pot_ET+Pot_Pc (4)

This resulted in 37 scoring maps with pixels labelled as DEF indicating that the water balance in that dekad contributes to the suitability or EXC indicating that the water balance in that dekad does not contribute to the suitability.

Step 4. Suitability maps per season. The suitability of a particular area and season for AWD is based on the proportion of DEF scores per season. This index ranges from 0 (no dekad in the season recording a DEF score) to 1 (all dekads in the season recording a DEF score). We set breakpoints for the DEF score index to separate classes of low suitability (LS), moderate suitability (MS) and high suitability (HS) conditions for AWD. In our “basic setting” setting these breakpoints were set in regular thirds, namely LS ranging from 0 to 0.33, MS from 0.34 to 0.66 and HS from 0.67 to 1.

This index is the basis for the seasonal suitability, but there is uncertainty as to what DEF score index indicates a climatically suitable area AWD. Here we address this uncertainty with a a second sensitivity analysis (Fig 1, Sensitivity Analysis 2) by defining a range of break points in the score index (Table 5) and computing the LS, MS and HS areas across them.

**Table 5:** Breakpoints on the DEF score index for the sensitivity analysis on low (LS), moderate (MS) and high (HS) suitability for AWD.

Breakpoints	Seasonal suitability for AWD based on DEF score		
	LS	MS	HS
1 (33-33-33, basic setting)	0.0–0.33	0.34–0.66	0.67–1.00
2 (20-60-20)	0.0–0.20	0.21–0.80	0.81–1.00
3 (25-50-25)	0.0–0.25	0.26–0.75	0.76–1.00
4 (30-40-30)	0.0–0.30	0.31–0.70	0.71–1.00

## Results and Discussion

Here we focus on the wet season results. Dry season results are reported only in S1 File, since more than 99% of the dry season rice area was deemed to be highly suitable under all combinations of Pot_Pc rates per texture class (Sensitivity Analysis 1, Table 4) and breakpoints (Sensitivity Analysis 2, Table 5) in the sensitivity analyses. The full set of sensitivity results for wet and dry seasons are reported in the Supporting Information (Figure A, Figure B, Figure C and Figure D in S1 File).

**Fig 6:**
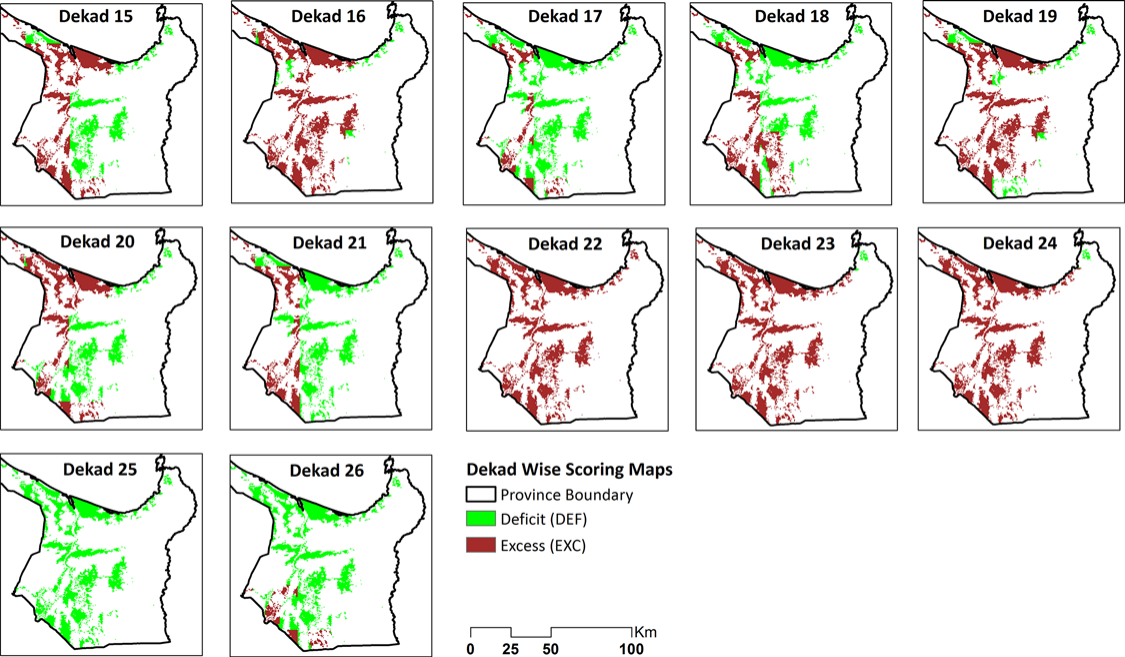
Dekad wise scoring maps in the wet season.

**Fig 7:**
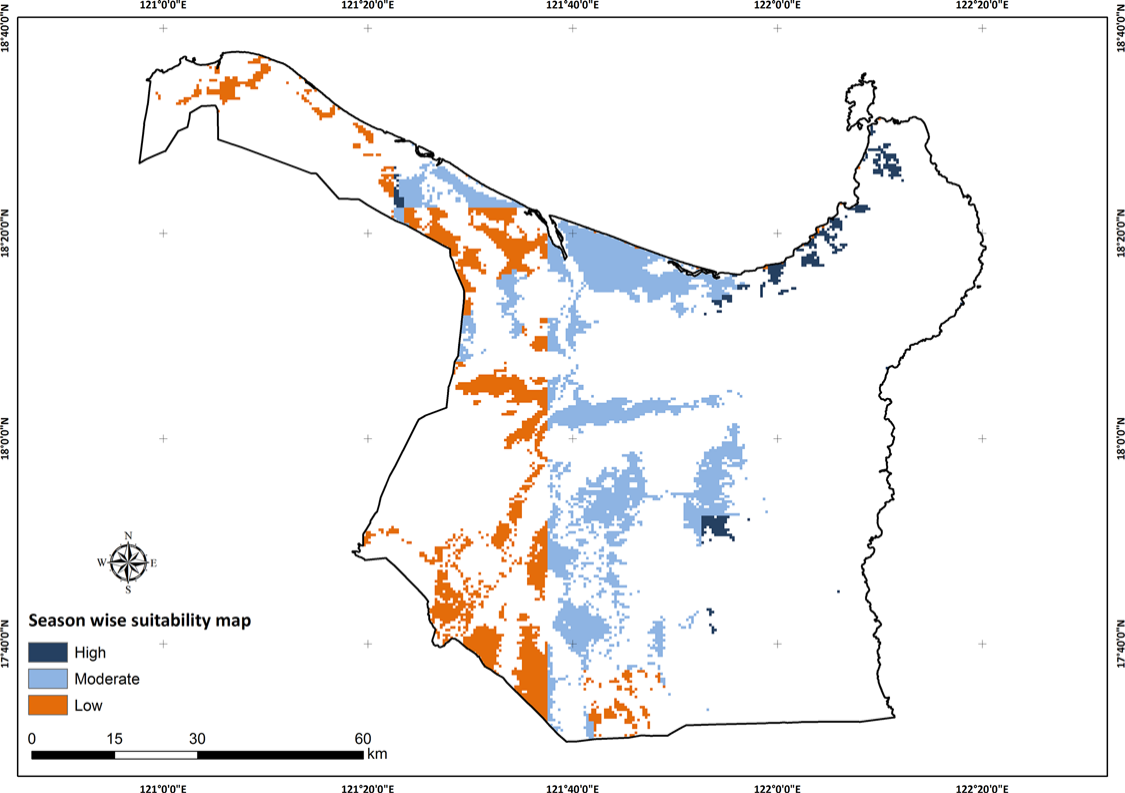
Suitability map for AWD in the wet season. Map shows the pixels identified as suitable for AWD in the wet season based on soil specific percolation rates (using the basic settings column in Table 4) and for suitability class breakpoints (using the basic settings row in Table 5).

**Fig 8:**
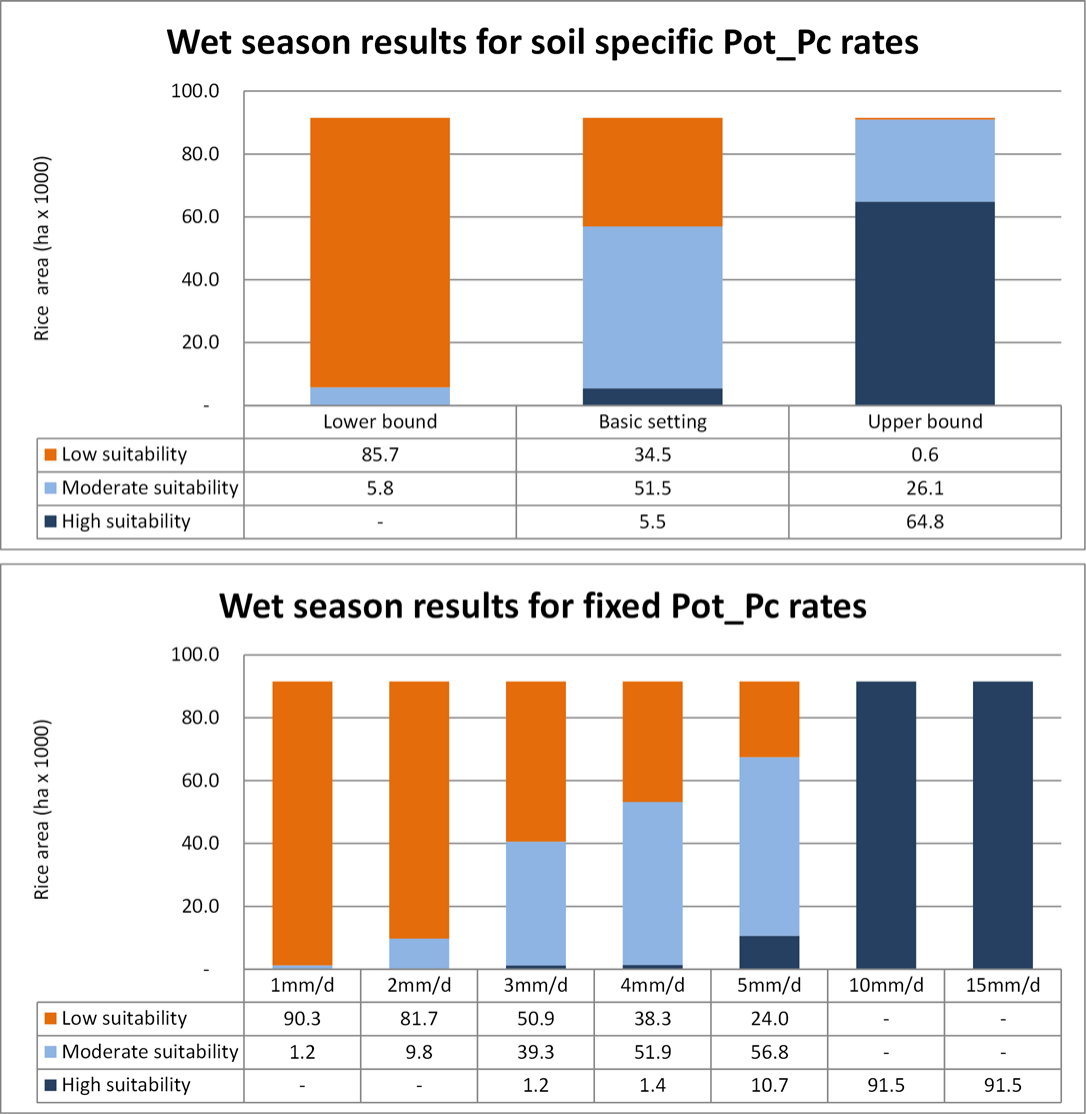
Sensitivity analysis on Pot_Pc for AWD in the wet season. Area of wet season rice (in hectares) deemed climatically suitable for AWD, by suitability class. The basic setting breakpoints were used for the suitability classes and each bar in the chart represents the suitable area for different Pot_Pc setting in the soil specific Pot_Pc rates (top panel) and fixed Pot_Pc rates (bottom panel).

The dekad scoring maps using the basic setting for soil specific Pot_Pc for the wet season (May to September) are shown in Fig 6. The seasonal suitability map for the wet season is shown in Fig 7 based on soil specific percolation rates (taken from the basic settings column of Table 4, Sensitivity Analysis 1) and the basic setting for suitability class breakpoints (taken from the basic settings row of Table 5, Sensitivity Analysis 2). Fig 8 shows the variation in area deemed suitable for AWD for different Pot_Pc settings or assumptions (Sensitivity Analysis 1) using the basic setting for the suitability class breakpoints (from row 1 of Table 5). The top panel of Fig 8 shows the suitable area for soil specific Pot_Pc rates and the bottom panel shows the results for fixed Pot_Pc rates.

The block like boundaries between different classes in the scoring maps (Fig 6) reflect the coarse spatial resolution of the rainfall and Pot_ET datasets. Despite this limitation, the sequence of maps still captures spatial and temporal variation from dekad to dekad through the season. Central and northern rice areas are regularly classed as DEF areas, especially at the start and end of the season, while the western rice growing areas are consistently classed as EXC except for the end of the season. The temporal patterns in DEF and EXC scores are driven by rainfall (Fig 3) which is low at the start of the season (onset of the monsoon), reaches a peak in dekads 22 to 24 (where consistent high rainfall results in EXC scores in almost all rice growing areas of the province (Fig 6) regardless of the spatial variability in Pot_Pc) and then reduces again towards the end of the season (recession of the monsoon). Pot_ET is consistent at 4–5 mm/d through the season and does not contribute substantially to the spatial or temporal patterns in this season (Fig 5).

The variability in the soil specific Pot_Pc is limited but does have some contribution (Fig 2, panel C). The two major SMUs (4413 and 4465) together occupy 69% of the province and both have a weighted Pot_Pc rates of 4.8 mm/d while SMU 4478 and 4589 in the central and northern parts of the province have lower rates (3.8 mm/d and 3.5 mm/d respectively) and areas (20% and 8% respectively). The effect of Pot_Pc can be seen especially in dekads 17 and 21 (Fig 6) where the spatial variability in Pot_Pc leads to more spatially detailed variations in the scoring than can be attributed to the coarse climate data.

The wet season suitability map for AWD (Fig 7) shows a clear east-west division between low and moderate/high suitability, with the north coast being largely moderately to highly suitable. This clearly reflects the accumulated dekad scores across the wet season where the eastern and northern areas received less rainfall through the season resulting in more frequent DEF scores. Highly suitable areas in the north east are the result of higher Pot_Pc values and lower rainfall. The most striking feature of this map is the large proportion wet season rice areas with moderate/high suitability under our climatic suitability assumptions. High suitability for the dry season was expected and was consistently present (Figures A, B, C and D in S1 File) under all permutations, since draining of the field is aided by low rainfall. However the presence of wet season suitability suggests that there is greater scope for AWD in relatively high rainfall areas than previously thought.

Fig 8 shows the rice area per suitability class summarized for the above case and for each Pot_Pc permutation within the same basic setting for breakpoints. From the soil specific basic setting dataset in the upper panel of Fig 8, 57,000 ha or 62% of the wet season rice area was deemed moderate or highly suitable while 34,500 ha were deemed to have low suitability. Comparable figures are shown in the 3mm/d, 4mm/d and 5mm/d Pot_Pc rates in the lower panel of Fig 8 (40,600 ha, 53,200 ha and 67,500 ha of moderate or high suitability respectively, ranging from 44% to 74% of the wet season rice area) since these fixed rates are comparable to the soil specific rates used in the basic setting (3.5 mm/d, 3.8 mm/d and 4.8 mm/d). The impact of selecting the very low or very high Pot_Pc rates is substantial with areas ranging from almost all low suitability (1 mm/d) to all high suitability (10 mm/d and 15 mm/d, Fig 8, bottom panel), though as described in Section 2.1.3, our choice of the lower and upper bounds in Pot_Pc (Fig 8, top panel) were deliberately inclusive of the most extreme values reported in the literature. The range of suitability is more constrained in the soil specific Pot_Pc but the trend is the same (ranging from 5,800 ha or 6% of moderate or high suitability for the lower bound to 90, 900 ha or 99% for the upper bound). The impact of different breakpoints in the suitability class (S1 File) is much less than the choice of Pot_Pc with the moderate or high suitability area ranging from 70,680 ha to 57,000 ha when using the basic setting for soil specific Pot_Pc.

The results of the sensitivity analysis in the dry season and the wet season suggest that the model is generating plausible results for climatic suitability to AWD when the best available information on soil permeability is used and the model performs as expected when extreme percolation rates are used.

## Conclusions

We have presented the first attempt of a spatial and temporal assessment of the climatic suitability for AWD. The assessment is based on a simple water balance model reflecting the possibility to drain or dry the rice field for a substantial duration of the rice season. The model relies on easily available and/or easily derived spatial and statistical data for rice areas, rice seasonality, rainfall, potential evapotranspiration, soil texture and percolation rates in rice soils. In cases where available information was not consistent (soil percolation rates) or non existent (definition of suitability classes) we provided plausible upper and lower bounds and explored the variability in suitable area estimation due to this uncertainty. The impact of selecting different percolation rates (within reasonable bounds) when estimating the area suitable for AWD was substantial. The lack of precise data on percolation rates is a limitation.

The climate suitability for AWD in the dry season was consistently high across all analyses, as would be expected for dry season rice systems that rely on irrigation. Additionally, a substantial area of the wet season rice was found to be climatically suitable for AWD, contradicting the notion that AWD is not suitable in the monsoon due to excessive rainfall that prevents drainage. Across Asia, it is possible that the climatically suitable area for AWD is larger than previously thought since over 70% of the annual rice area in Asia is planted in the wet season. This potential can be assessed on a case by case basis across different countries, rice systems and management practices. However in the wet season the ground water levels in valleys often rises such that the percolation rate becomes nil. The temporal dynamics percolation rates are not accounted for in the current approach, but could be introduced since the assessment is based on a dekadal timestep.

This method provides a framework for assessing AWD suitability across other regions and countries. Estimations of the global rice area that could be suitable for AWD using this approach would give a plausible upper bound to the potential water savings of the technology as well as potential methane emission reductions, both of which are currently unknown and both of which could be substantial considering the global total of 100m ha of irrigated rice. Beyond that, with finer level climate data, and additional context-specific information on irrigation facilities and water pricing, the approach could be the basis of dissemination programs and appropriate extension service training for AWD.

## Supporting Information

S1 File Sensitivity analysis on Pot_Pc for AWD in the wet and dry season. Area of wet season and dry season rice (in hectares) deemed climatically suitable for AWD, by suitability class for soil specific Pot_Pc rates and fixed Pot_Pc rates. **Figure A** shows the results using breakpoint setting 1 (33-33-33, basic setting). **Figure B** shows the results using breakpoint setting 2 (20-60-20), **Figure C** shows the results using breakpoint setting 3 (25-50-25) and, **Figure D** shows the results using breakpoint setting 4 (30-40-40).
(DOCX)
