# Supplementary material for: Climate-Determined Suitability of the Water Saving Technology "Alternate Wetting and Drying" in Rice Systems: A Scalable Methodology demonstrated for a Province in the Philippines
Source: PLoS One. 2015 Dec 21;10(12):e0145268. doi: 10.1371/journal.pone.0145268 (PMC4686430; doi:10.1371/journal.pone.0145268)
Supplement: S1 File — Area of wet season and dry season rice (in hectares) deemed climatically suitable for AWD, by suitability class for soil specific Pot_Pc rates and fixed Pot_Pc rates. Figure A shows the results using breakpoint setting 1 (33-33-33, basic setting). Figure B shows the results using breakpoint setting 2 (20-60-20), Figure C shows the results using breakpoint setting 3 (25-50-25) and, Figure D shows the results using breakpoint setting 4 (30-40-40). (DOCX) [file pone.0145268.s001.docx]

**S1 FILE**  **Sensitivity analysis on Pot_Pc for AWD in the wet and dry season.** Area of wet season and dry season rice (in hectares) deemed climatically suitable for AWD, by suitability class for soil specific Pot_Pc rates and fixed Pot_Pc rates. **Figure A** shows the results using breakpoint setting 1 (33-33-33, basic setting). Figure B shows the results using breakpoint setting 2 (20-60-20), **Figure C** shows the results using breakpoint setting 3 (25-50-25) and, **Figure D** shows the results using breakpoint setting 4 (30-40-40).

**Figure A.**

**Figure B.**

**Figure C.**

**Figure D.**
